# Traumatic posterior atlantooccipital dislocation combined with type II dens fracture and C1 anterior arch fracture: Two case reports

**DOI:** 10.1097/MD.0000000000017666

**Published:** 2019-10-25

**Authors:** Dong-Gune Chang, Jong-Beom Park, Yoon Joo Cho, Gang-Un Kim

**Affiliations:** a Department of Orthopaedic Surgery, Sanggye Paik Hospital, College of Medicine, Inje University, Seoul,; b Department of Orthopaedic Surgery, Uijeongbu St. Mary's Hospital, The Catholic University of Korea, South Korea.

**Keywords:** atlantooccipital dislocation, C1 anterior arch fracture, craniocervical instability, transverse atlantal ligament, type II dens fracture, upper cervical spine

## Abstract

**Rationale:**

Traumatic AOD is rare but highly associated with upper cervical spine injuries. We found no references in the literature of traumatic posterior atlantooccipital dislocation (AOD) combined with type II dens fracture (Anderson-D’Alonzo classification) and C1 anterior arch fracture.

**Patient concerns:**

The first case was a 93-year-old male patient who was admitted to the Emergency Department complaining of incomplete quadriplegia after a fall from a height. The second was a 53-year-old male patient who visited the emergency department complaining of posterior neck pain following a high-speed motor vehicle collision.

**Diagnosis:**

Reconstructed computed tomography (CT) scans clearly demonstrated posterior AOD combined with type II dens fracture and C1 anterior arch fracture. In addition, magnetic resonance imaging (MRI) also revealed type II transverse atlantal ligament injury (Dickman's classification) in the first patient.

**Interventions:**

The patients chose not to undergo surgery; instead, they were immobilized with a rigid cervical brace.

**Outcomes:**

The patients were lost to follow-up.

**Lessons:**

A thorough clinical evaluation and radiologic investigation (CT and MRI) on concomitant upper cervical injuries should be evaluated in traumatic AOD patients.

## 1 Introduction

Atlantooccipital dislocation (AOD) is a rare but highly associated with upper cervical spine injuries and usually carries a fatal prognosis. Because AOD often results in instability of the occipitocervical junction, dislocation leads to compression of neurologic structures at the pontomedullary junction followed by the sudden death of the patient. Moreover, the injury is typically associated with intracranial hemorrhaging.^[1–3]^ Thus, immediate rapid diagnosis and appropriate management are essential to prevent subsequent neurologic deterioration or death.^[4,5]^

AOD occurs as either an isolated injury or as a part of combined trauma. Previous reports have indicated its association with occipital, Jefferson, or type I dens fracture.^[6,7]^ However, the initial diagnosis is often overlooked; therefore, careful suspicion and a thorough evaluation of the upper cervical spine should be made to avoid missing the diagnosis of AOD.^[8]^

In the current study, the authors present the first 2 cases of radiographs showing traumatic posterior AOD combined with type II dens fracture and C1 anterior arch fracture, which have not been reported previously.

## 2 Case report

### 2.1 Case 1

**Figure 1:**
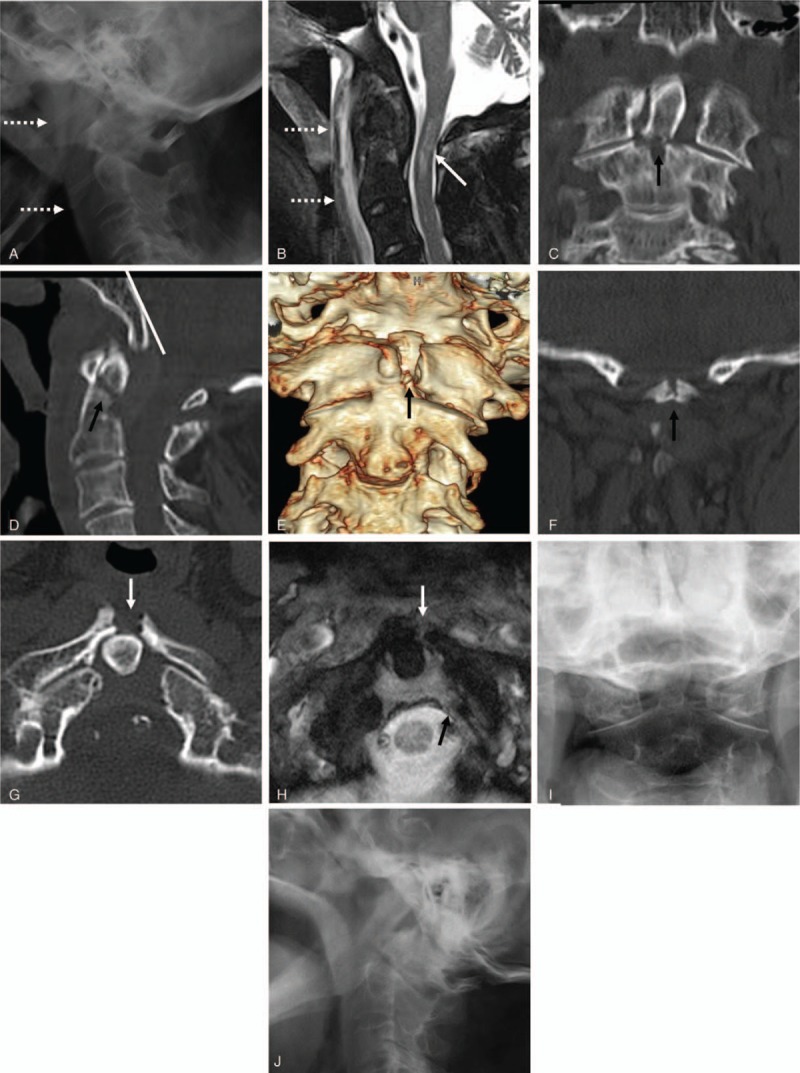
Initial lateral radiographs of the cervical spine (A) and sagittal MRI (B) showing increased anterior soft tissue swelling due to retropharyngeal hematoma (white dotted arrows) and intramedullary hemorrhage (white arrow). Coronal (C) and sagittal (D) reconstructed CT scans demonstrated running of the Wackenheim line (white line) behind the tip of the dens, indicating posterior AOD and type II dens fracture (Anderson-D’Alonzo classification) (black arrows). Three-dimensional (E) and coronal (F) reconstructed CT scans showing a midline sagittal split fracture of the C1 anterior arch (white arrows). Axial CT scan (G) and MRI (H) revealed a midline sagittal split fracture of the C1 anterior arch (white arrow) and a type II TAL injury according to Dickman's classification. Three months following conservative treatment, the posterior AOD and type II dens fracture were posteriorly displaced compared to the initial presentation (I and J). AOD = atlantooccipital dislocation, CT = computed tomography, MRI = magnetic resonance imaging.

A 93-year-old male was admitted to the Emergency Department complaining of incomplete quadriplegia after a fall from a height. A soft-collar brace was immediately placed on the patient, and plain radiographs of the cervical spine were obtained. Initial lateral radiographs of the cervical spine (Fig. 1A) and sagittal magnetic resonance imaging (MRI) (Fig. 1B) revealed increased anterior soft tissue swelling due to retropharyngeal hematoma and intramedullary hemorrhaging. Coronal and sagittal reconstructed computed tomography (CT) scans (Fig. 1C and D) showed type II dens fracture (Anderson-D’Alonzo classification) and running of the Wackenheim line behind the dens, indicating posterior AOD. Three-dimensional, coronal, and axial CT scans showed a midline sagittal split fracture of the C1 anterior arch and type II fracture of the dens (Anderson-D’Alonzo classification) (Fig. 1E–G). Furthermore, axial MRI revealed type II transverse atlantal ligament (TAL) injury according to Dickman's classification (Fig. 1H). Because the patient was too old to safely undergo surgical treatment, he and his family did not opt for surgery. Instead, the patient was treated with the application of a rigid cervical brace for three months (Fig. 1I and J), and then he was lost to follow-up.

### 2.2 Case 2

**Figure 2:**
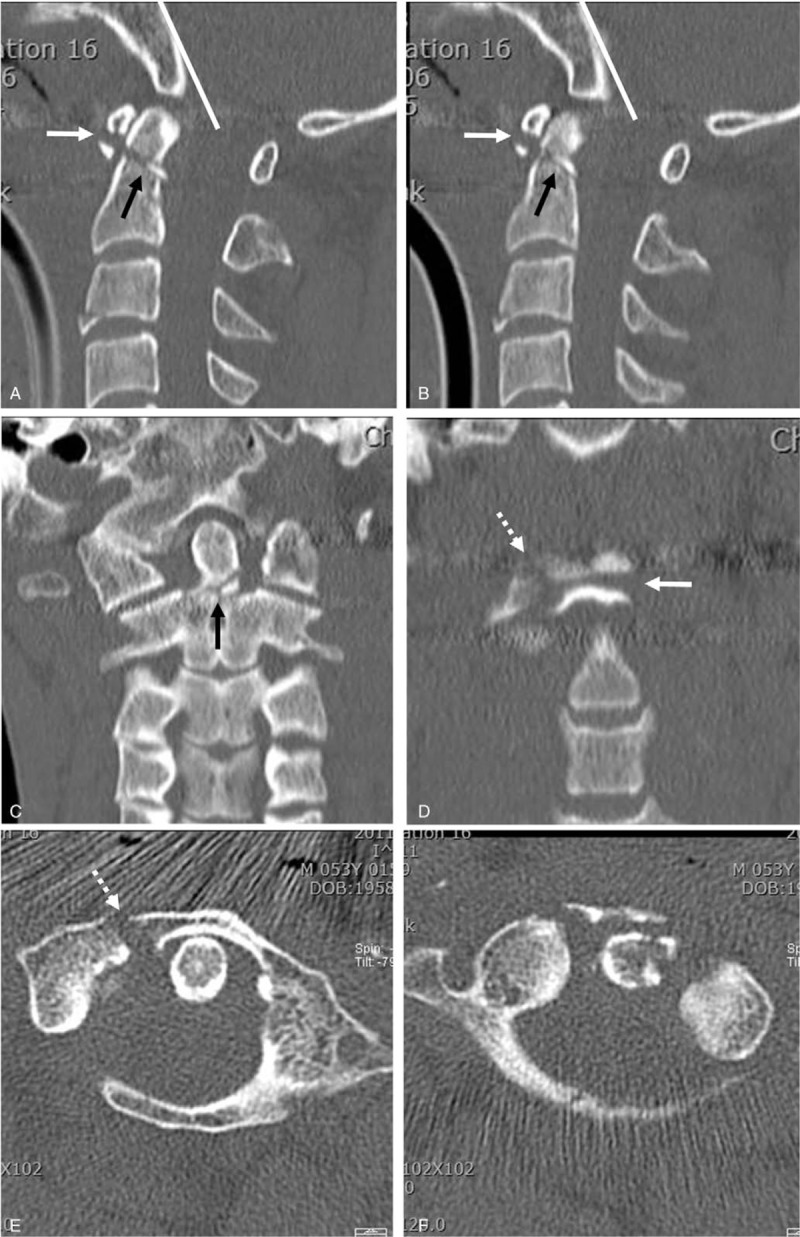
Sagittal (A and B) reconstructed CT scans showing running of the Wackenheim line (white line) behind the dens, indicating posterior atlantooccipital dislocation, dens type II fracture with comminution (Anderson - D’Alonzo classification) (black arrow), and fracture of the C1 anterior arch. Coronal (C and D) reconstructed CT scans revealed dens type II fracture with comminution and widening of the right atlantodental interval. Axial (E and F) CT scans showed a sagittal split fracture of C1 (white dot arrow) with a small avulsion fragment from a right lateral mass of C1, suggestive of a type II transverse atlantal ligament injury. CT = computed tomography.

A 53-year-old male visited our emergency department after a high-speed motor vehicle collision. He complained of posterior neck pain, but motor and sensory functions of the upper and lower extremities showed no abnormalities. Sagittal reconstructed CT scans (Fig. 2A and B) revealed running of the Wackenheim line behind the dens, indicating posterior AOD and dens type II fracture with comminution (Anderson-D’Alonzo classification). Type II dens fracture with comminution and a combined horizontal and sagittal split fracture of the C1 anterior arch were shown in the coronal (Fig. 2C and D) and axial (Fig. 2E and F) reconstructed CT scans, respectively. In addition, an axial CT scan (Fig. 2E) indicated a widened right atlantodental interval with an avulsion fragment from the lateral mass of C1, which is suggestive of a type II TAL injury. We planned to perform occipitocervical fusion; however, the patient refused surgical treatment. He was immobilized with a rigid cervical brace, transferred to another hospital near his home, and then lost to follow-up.

## 3 Discussion

Traumatic AOD is a serious injury. It is often related to intracranial hemorrhaging and critical compression of the pontomedullary junction.^[3,9]^ AOD is usually caused by a complete rupture of the ligamentous structures of the upper cervical spine, including the tectorial membrane, apical ligament, and alar ligament, by excessive hyperextension or hyperflexion of the head-neck junction.^[2,10,11]^ Since Blackwood^[12]^ first reported the condition in 1908, most traumatic AOD cases have been of the anterior type; however, posterior,^[13]^ lateral,^[14]^ and longitudinal^[15]^ dislocations have been rarely reported.

Related injuries of the upper cervical spine that have occurred in conjunction with AOD are occipital condyle fracture, Jefferson fracture,^[7]^ and type I dens fracture.^[6]^ Survival after traumatic AOD with unilateral or bilateral type I dens fracture has also been reported.^[6,13]^ However, we found no references in the literature of a combined lesion of AOD with the coexistence of type II dens fracture and C1 anterior arch fracture. Our report is the first study to describe the coexistence of a type II dens fracture and a C1 anterior arch fracture in posterior AOD. About 50% of AOD cases are missed after injuries to the cervical spine.^[8,16]^ Therefore, if a type II dens fracture is found in the initial patient assessment, evaluation of any accompanying craniocervical instability should be carried out.

The treatment of choice for AOD is surgical stabilization of the upper cervical spine using occipitocervical fusion because complete healing of the torn ligamentous structures is difficult to achieve with conservative care, and the risk of redisplacement with subsequent compression of neural structures always exists.^[17,18]^ However, our cases underwent conservative treatment without surgical management because the patients did not want to undergo surgery. Additionally, the first patient was extremely elderly; patients over age 90 are considered to have a high risk of morbidity for the surgical treatment.^[19,20]^

Dickman classified TAL injuries into two types; a type I TAL injury occurs when there is pure ligamentous disruption in its midportion or at its periosteal insertion, while type II injury shows an avulsion fracture or disconnection of the tubercle for the insertion of the TAL from the C1 lateral mass in a comminuted C1 lateral mass.^[21]^ In our experience, both of our cases demonstrated a type II TAL injury, and they were confirmed by MRI and CT, respectively.

Our cases represented 2 types of C1 anterior arch fracture with type II dens fracture. The fracture pattern in case 1 showed only the sagittal split type accompanied by massive retrodental hematoma and instability of the occipito-cervical junction, resulting in quadriplegia due to spinal cord injury. However, case 2 presented with combined horizontal and sagittal split fractures of the C1 anterior arch, and he only complained of posterior neck pain; no neurologic deficits were evident. As mentioned in a previous article,^[6]^ the appearance of a C1 fracture rather than the dens type of fracture seems to contribute more to craniocervical instability. In all reported cases to date, horizontal fractures of the anterior arch of C1 have not been associated with dens fractures.^[22–25]^ However, our case showed features that were contrary to previous reports.

In conclusion, our patients were the first reported cases of traumatic posterior AOD combined with type II dens fracture and C1 anterior arch fracture that survived the injury. Therefore, a thorough clinical evaluation and radiologic investigation (CT and MRI) on concomitant upper cervical injuries should be evaluated in traumatic AOD.

## Author contributions

**Conceptualization:** Dong-Gune Chang, Jong-Beom Park.

**Investigation:** Yoon Joo Cho.

**Methodology:** Jong-Beom Park, Yoon Joo Cho.

**Project administration:** Jong-Beom Park.

**Supervision:** Jong-Beom Park.

**Validation:** Dong-Gune Chang.

**Visualization:** Jong-Beom Park.

**Writing – original draft:** Dong-Gune Chang, Gang-Un Kim.

**Writing – review & editing:** Dong-Gune Chang.
